# Reviewed article: Luis MA, Mota JC, D’Oliveira CAFB, Vasconcelos CH, Silva RP, Junior ABD, Regina FL, Lima CM, Sá NB, Cardoso LO. Factors associated with transgender people mortality due to violent causes: a cross-sectional study, Brazil, 2014-2022. Epidemiol Serv Saude. 2026:35;e40748

**DOI:** 10.1590/S2237-96222026v35e20240748.a

**Published:** 2026-03-16

**Authors:** Danilo Martins

**Affiliations:** 1 Universidade de Pernambuco, Recife, PE, Brazil

## Peer review A

## Questionnaire

Does the manuscript contain new and significant information to justify publication? Yes

Does the Abstract (Summary) clearly and accurately describe the content of the article? No

Is the problem significant and concisely stated? No

Are the methods described comprehensively? No

Are the interpretations and conclusions justified by the results? No

Is adequate reference made to other work in the field? Yes

## Manuscript Structure

Length of article is: Adequate

Number of tables is: Adequate

Number of figures is: To few

Please state any conflict(s) of interest that you have in relation to the review of this paper. None

Interest: Good 

Quality: Below Average

Originality: Average

Overall: Average

Recommendation: Major Revision

## Comments

A introdução do artigo destaca o problema da violência contra pessoas trans no Brasil, contextualizando-o em relação a dados globais e locais. Há um foco na importância de políticas públicas e no avanço da coleta de dados, como o registro de identidade de gênero no Sistema de Informação de Agravos de Notificação (Sinan). O SIM já inclui o quesito identidade de gênero e que possibilita análises como esta? O problema de invisibilidade e a necessidade de melhor entendimento sobre mortalidade são abordados de forma compreensível, porém, superficialmente. Contextualiza bem o problema da violência e a relevância do estudo. Apresenta dados recentes para justificar a relevância do tema. Há menção de fatores estruturais e políticos na introdução, mas a conexão entre esses fatores e o objetivo do estudo poderia ser mais explícita. Cadê a pergunta de pesquisa? Sugiro definir o que são “fatores associados”, ainda na introdução, e adequar o título e objetivo para “fatores associados à mortalidade por causas violentas entre pessoas trans no Brasil durante os anos de 2014 a 2022” e “identificar fatores associados à mortalidade por causas violentas entre pessoas trans no Brasil durante os anos de 2014 a 2022”, respectivamente. A metodologia é detalhada, descrevendo o estudo transversal baseado em pareamento determinístico e probabilístico entre as bases Sinan e SIM. As variáveis consideradas incluem identidade de gênero, tipo de violência e tempo entre notificação e óbito. A abordagem estatística é adequada para o objetivo. Os métodos de pareamento e as ferramentas utilizadas (e.g., RecordLinkage) devem ser melhor descritos. Apesar de a introdução destacar aspectos estruturais da violência, a metodologia cita variáveis contextuais, como fatores socioeconômicos, que podem contribuir para uma análise mais profunda. A ausência de justificativa mais robusta para a escolha do período temporal analisado (2014-2022) pode limitar a compreensão dos leitores sobre o recorte temporal. Os resultados apresentam dados relevantes, como o aumento das notificações de violência e a maior prevalência de óbitos em grupos jovens e em situações de lesão autoprovocada. A análise estatística inclui razões de prevalência ajustadas, o que fortalece a confiabilidade dos achados. Não há uma análise exploratória de outros fatores estruturais, como acesso a serviços de saúde ou impacto de políticas públicas mencionadas na introdução. Os resultados sobre aumento de notificações ao longo do tempo são apresentados sem uma análise crítica da influência de melhorias nos sistemas de registro. A discussão conecta os resultados ao contexto global e local da violência contra pessoas trans. Políticas públicas e desigualdades estruturais são mencionadas como fatores subjacentes. A discussão contextualiza os achados no âmbito global e regional, destacando particularidades do Brasil. Faz boas conexões com estudos anteriores. Aspectos estruturais precisam ser melhor discutidos (e.g., desemprego, educação). Embora o artigo enfatize a importância de ações políticas e sociais, não há sugestão de medidas específicas baseadas nos achados do estudo. A conclusão reforça a gravidade da violência contra pessoas trans e a necessidade de políticas públicas inclusivas. A conclusão extrapola os achados ao propor medidas amplas sem base concreta nos resultados apresentados. O artigo apresenta um problema relevante e utiliza uma metodologia sólida para abordar parte do objetivo. No entanto, há inconsistências entre introdução, metodologia e discussão, especialmente em relação à análise de fatores estruturais. Recomenda-se: Melhor alinhamento entre os elementos principais do artigo. Exploração mais detalhada de variáveis contextuais na metodologia/resultados e discussões. Discussão mais focada nos resultados concretos do estudo.

